# Dry Electrodes for Surface Electromyography Based on Architectured Titanium Thin Films

**DOI:** 10.3390/ma13092135

**Published:** 2020-05-05

**Authors:** Marco S. Rodrigues, Patrique Fiedler, Nora Küchler, Rui P. Domingues, Cláudia Lopes, Joel Borges, Jens Haueisen, Filipe Vaz

**Affiliations:** 1 Centro de Física, Universidade do Minho, Campus de Gualtar, 4710-057 Braga, Portugal; marcopsr@gmail.com (M.S.R.); ruigojasdomingues@gmail.com (R.P.D.); joelborges@fisica.uminho.pt (J.B.); fvaz@fisica.uminho.pt (F.V.); 2 Institute of Biomedical Engineering and Informatics, Technische Universität Ilmenau, 98693 Ilmenau, Germany; patrique.fiedler@tu-ilmenau.de (P.F.); nora.kuechler@tu-ilmenau.de (N.K.); jens.haueisen@tu-ilmenau.de (J.H.); 3 eemagine Medical Imaging Solutions GmbH, 10243 Berlin, Germany; 4 Biomagnetic Center, Department of Neurology, University Hospital Jena, 07747 Jena, Germany

**Keywords:** biopotential electrodes, dry electrodes, electromyography, thin films, magnetron sputtering, glancing angle deposition

## Abstract

Electrodes of silver/silver chloride (Ag/AgCl) are dominant in clinical settings for surface electromyography (sEMG) recordings. These electrodes need a conductive electrolyte gel to ensure proper performance, which dries during long-term measurements inhibiting the immediate electrode’s reuse and is often linked to skin irritation episodes. To overcome these drawbacks, a new type of dry electrodes based on architectured titanium (Ti) thin films were proposed in this work. The architectured microstructures were zigzags, obtained with different sputtering incidence angles (α), which have been shown to directly influence the films’ porosity and electrical conductivity. The electrodes were prepared using thermoplastic polyurethane (TPU) and stainless-steel (SS) substrates, and their performance was tested in male volunteers (athletes) by recording electromyography (EMG) signals, preceded by electrode-skin impedance measurements. In general, the results showed that both SS and TPU dry electrodes can be used for sEMG recordings. While SS electrodes almost match the signal quality parameters of reference electrodes of Ag/AgCl, the performance of electrodes based on TPU functionalized with a Ti thin film still requires further improvements. Noteworthy was the clear increase of the signal to noise ratios when the thin films’ microstructure evolved from normal growth towards zigzag microstructures, meaning that further tailoring of the thin film microstructure is a possible route to achieve optimized performances. Finally, the developed dry electrodes are reusable and allow for multiple EMG recordings without being replaced.

## 1. Introduction

Biopotential measurements including electromyography (EMG), electroencephalography (EEG), and electrocardiography (ECG) play a key role in medical research and clinical routines, being essential for the study, strategic prevention, early diagnosis, and therapy of neurological and neuromuscular disorders [1]. EMG provides crucial additional information about nerve and muscle function not accessible using other modalities or on the sole basis of a patient’s clinical history. In case of a muscle dysfunction, an EMG recording supports the detection of the location and extension of its pathological origin. Besides basic biomedical and physiological studies, EMG is also a tool for applied medical research, physiotherapy, rehabilitation, ergonomics, and sports science [2,3]. Surface electromyography (sEMG) is a non-invasive EMG method using electrodes applied to the body surface, enabling both the study of muscle function in naturalistic setups and the high-density mapping of nerve–muscle interactions during complex movements. EMG therefore supports an increasing understanding and reliability of related medical diagnostics and therapy.

This work is devoted to the development of a new physiological monitoring system based on thin film dry electrodes for the recording of sEMG signals in different environments/settings and conditions. The force development of a muscle involves several steps. During low strain, only motor units with slow muscle fibers are activated. With increasing strain, the frequency of the action potentials of those muscle fibers increases and additional motor units are recruited. Therefore, the force of the muscle contraction is characterized by the number of involved axons and by the frequency of the action potentials of every axon. sEMG records changes of the body surface potentials caused by the superimposed action potentials on an underlying, contracting muscle [4].

For an accurate, reliable, and reproducible sEMG measurement, several factors need to be considered, namely: (a) avoid noisy electrical environments [5]; (b) minimize artefacts caused by changes of the electrode–skin interface or by cable movements [6]; (c) consider the biological conditions (e.g., skin condition, body temperature, movement) [7]; and (d) exact positioning of the electrodes according to international standards and/or conventions [8]. In fact, the variety of inter- and intraindividual differences in tissue and body condition, movement execution, and physiological or pathological changes inevitably increases the variability of sEMG results. Of paramount importance is also the set of electrodes used for EMG acquisition, which must be carefully selected considering not only the application setup, the quality of the signal and potential frequency band, but also application time and the patient’s skin sensitivity.

Traditionally, wet Ag/AgCl electrodes are considered the gold standard for biopotential measurements. These electrodes are non-polarizable, show a low and almost frequency independent electrode–skin interface impedance, in the order of a few tens of kΩ [9,10]. Nevertheless, these electrodes need an electrolyte gel to ensure a reliable electrode–skin connection in combination with an adhesive patch to fix the electrode position, thereby limiting gel spreading. Together with the adhesives and the glues of the setup, the need to use a gel to reduce the electrode–skin impedance often promotes contact dermatitis. Moreover, the gel easily dries out during long time acquisitions, which distorts the recorded signal.

In this context, dry electrodes have been proposed as an alternative to replace the Ag/AgCl electrodes [5,11]. Most metals provide excellent fabrication stability, good mechanical and physical properties, and are suitable for mass production [12]. For example, stainless steel (SS) is commonly used as a substrate for dry electrodes in consumer products for sEMG and ECG [13,14]. However, purely metallic electrodes pose limitations in terms of adaptivity to (changing) body curvature and integration in clothing. Electrodes based on flexible materials (e.g., polymers) tackle these limitations as they ensure better wearing comfort for the patient, since the electrode surface aligns to the curvature of the body shape and therefore reduces motion artefacts [15] during long-term sEMG. Polymers such as polysiloxane [16,17], polyurethane [18], and parylene [19] have previously been used as substrate materials. Another relevant example is thermoplastic polyurethane (TPU), which is a biocompatible thermoplastic characterized by its excellent balance between mechanical properties (high flexibility), chemical barrier behavior, and soft tact [9]. Because of these characteristics, it is often used for implantable devices [20] and has been previously used as a substrate in bioelectrodes [9]. Furthermore, it is possible to use TPU as a material in 3D printing, allowing to design it according to the desired requirements and to be applied for mass production. Nevertheless, since it is an electrically insulating material, it must be functionalized with a conductive layer such as a metallic thin film, able to follow up its flexibility.

The deposited thin film itself has to meet several requirements to be considered suitable for biomedical devices. Special properties such as high corrosion and wear resistance, good mechanical ductility, as well as chemical and thermal stability are required. Additionally, the thin film material should be biocompatible and capable of preventing fungus, yeast, and bacteria proliferation, existing in body fluids like human sweat [21]. Since titanium (Ti) meets these requirements, it is used in structural and functional applications, especially in biomedical industries [22,23].

Depending on the microstructural characteristics, like morphology, textures, and porosity, the properties of Ti thin films, produced by physical vapor deposition (PVD) techniques are extremely variable and tunable [24,25]. Among PVD techniques, magnetron sputtering with glancing/oblique angle deposition (GLAD/OAD) allows the adjustment of conventional columnar metallic thin films, into different growth architectures (inclined columns, zig-zags, spirals, etc.) [26].

Using the GLAD deposition process, the sputtered atoms from the target arrive at the substrate surface at an incidence angle α and cause random distribution of nuclei. As more atoms arrive to the substrate, the nuclei start growing into columns. The shadows of taller columns suppress the further growth of their smaller neighbors. Eventually, some of the columns stop growing completely, being fully shadowed by their tall neighbors [26]. When the substrate holder normal axis is pointed directly at the target, an α angle of 0° occurs. When the substrate holder’s normal axis is perpendicular to the target, an α angle of 90° occurs. The arising architectures have a major effect on the mechanical and electrical behavior of the thin films.

The aim of this work was the deposition of Ti thin films onto TPU and SS substrates by magnetron sputtering with different nanostructures. Oblique angle deposition was used to create zig-zag structures with tailored physical properties and improved electrode performance for sEMG acquisitions. Signal-to-noise ratio (SNR) and power spectral density (PSD) of sEMG recordings, acquired in-vivo during a multi-volunteer study, were employed to evaluate the electrodes’ signal quality. The electrode–skin impedance was also evaluated as it may influence the signal quality of the dry electrodes depending on the used biosignal amplifier.

## 2. Materials and Methods

### 2.1. Preparation and Characterization of the Dry Electrodes

**Figure 1:**
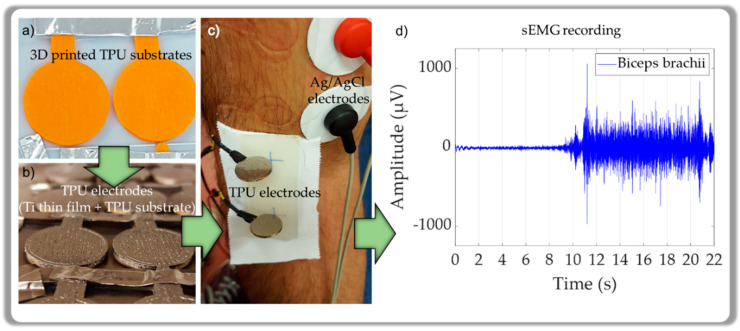
Thermoplastic polyurethane (TPU) electrode after: (**a**) 3D printing process, (**b**) thin film deposition, and (**c**) surface electromyography (sEMG) measurements; (**d**) exemplifies an sEMG recording from the Biceps brachii muscle.

For biosignal acquisition, the thin films were deposited onto SS 316 (Global Metals, Victoria, Australia) and TPU (Filaflex, Recreus, Alicante, Spain) disk-shaped substrates (Figure 1a), with 16 mm diameter and thicknesses of 1.0 mm and 1.5 mm, respectively. As reference electrodes, Ag/AgCl electrodes (SNAP Euro ECG electrodes, FIAB, Firenze, Italy) were used. Silicon substrates with (100) orientation (Boron doped p-type) were used for chemical, morphological, and structural characterization.

Before the depositions, both SS and TPU substrates were cleaned with ethanol. To remove possible molecular layers of contaminants from the sample surfaces and to increase hydrophilicity, the substrates were plasma treated [9]. For this, a Diener low pressure plasma (base pressure of 20 Pa) equipment with a 40 kHz RF generator (Zepto Model, Diener Electronic, Ebhausen, Germany) was used. For TPU, the activation was performed with Ar plasma for 15 min, which was found to be the most efficient treatment for this polymer [9].

The Ti thin films with zigzag microstructures were prepared by DC magnetron sputtering in a custom-made deposition system (base pressure of 3 × 10^−4^ Pa), equipped with a glancing angle deposition holder [27]. A rectangular titanium target (200 × 100 × 6 mm^3^) with 99.99% purity was used as cathode, and the plasma was generated with a current density of 75 A.m^−2^ in an Ar atmosphere with a pressure of 3.5 × 10^−1^ Pa. Figure 1b shows the TPU polymer after thin film deposition.

The main parameter studied was the incidence angle α of the flux of sputtered atoms impinging the substrate in order to obtain two full zigzag structures (two periods). The angles used were 0°, 40°, 60°, and 80°. Further details about the GLAD system can be found elsewhere [27].

The microstructural characteristics of the thin films were analyzed by Scanning Electron Microscopy (SEM) using a Dual Beam SEM/FIB FEI Helios 600i (Thermo Fisher SCIENTIFIC, Waltham, MA, USA). The resulting micrographs were analyzed with MATLAB (version R2018a, The MathWorks, Inc., Natick, MA, USA) [28]. The films were also characterized by X-ray diffraction (XRD) using a Bruker D8 (Bruker, Billerica, MA, USA) focus diffractometer with a cobalt X-ray tube (Co-Kα1, λ = 1.78897 Å) in a θ/2θ configuration with a step of 0.02° per 0.2 s and a 2θ angle ranging from 20° to 80°.

The electrical resistivity was measured with a custom-made system using the four-point probe method, in van-der-Pauw geometry [29].

### 2.2. Signal Acquisition and Processing Using the Dry Electrodes vs. Conventional Ag/AgCl Electrodes

The electrodes were validated with the collaboration of twenty professional handball players’ volunteers. Each volunteer gave his written consent for the data recording and processing in accordance with local privacy protection laws. All three electrode types (two dry, SS and TPU, and one set of conventional electrodes) were tested in male volunteers between the ages of 19 and 28 who exercise their arm muscles at least 4 times a week. One set of dry electrodes were re-used in all volunteers (with the proper cleaning steps), while the reference electrodes of Ag/AgCl were disposable ones (one set per volunteer).

In order to minimize the systematic influence, the electrode test sequence was randomized for each volunteer. The electrodes were placed on the biceps brachii of the volunteers’ dominant arm. The volunteer was seated on a chair with the elbow flexed at slightly obtuse angle. Before each acquisition, the volunteer’s skin was cleaned with 70% (v/v) ethyl alcohol. After the alcohol was vaporized and the skin dried, the electrodes were placed. The dry electrodes were mounted together on a piece of silicone on which the 20 mm center distance of the electrodes was marked. This assembly was tightened to the skin with tape (Strappal, BSN Medical, Luxembourg). The electrodes were placed in the direction of the line between the medial acromion and the fossa cubit at 1/3 from the fossa cubit, according to the Surface Electromyography for the Non-Invasive Assessment of Muscles (SENIAM) project recommendations [8]. The patient ground was located on the bony prominences of the wrist.

Before the acquisitions, the maximum voluntary contraction of the volunteers’ dominant arm was assessed.

**Figure 2:**
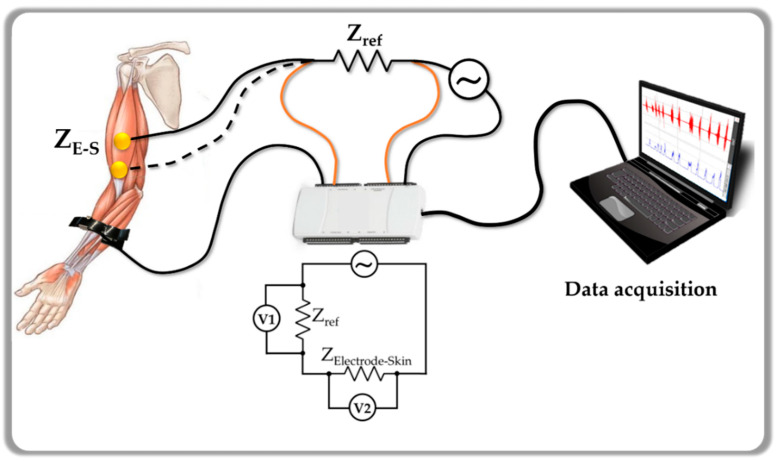
Scheme of the portable device setup used to measure the electrode-skin impedance, prior to the sEMG recording.

The electrode-skin impedance was measured at 10 Hz, 50 Hz, 100 Hz, 500 Hz, and 1000 Hz for each pair of electrodes, which includes the most important frequency range for sEMG (10 Hz to 500 Hz) [30]. The measurements were made using a NI USB-6216 Isolated Multifunction I/O (National Instruments, Austin, TX, USA) and LabView (National Instruments, Austin, TX, USA) environment. The setup is outlined in Figure 2 and uses a known voltage V_1_ to measure V_2_. The absolute electrode–skin impedance value (Z_E-S_) can be determined using Equation (1), with a known impedance value (Z_ref_). This portable impedance meter was calibrated using a HP 4192A LF impedance analyzer (Hewlett Packard Company, Palo Alto, CA, USA). The values were evaluated with MATLAB software (version R2018a, The MathWorks, Inc., Natick, MA, USA).

(1) $$\frac{V1}{V2}=1+\frac{Z_{ref}}{Z_{E-S}}$$

After the impedance measurements, the EMG signal was recorded (Figure 1c) during 10 s of the volunteers resting state followed by 10 s of physical strain (see example of Figure 1d). The sEMG was recorded with 20% and 40% of the maximum voluntary contraction (load20 and load40). For the acquisition of the electromyogram an eego mini amplifier (EE-410, ANT neuro, Hengelo, The Netherlands) was used.

The raw signal was processed using an adapted and custom MATLAB code [31,32]. For the signal processing, only 5 s of both the resting state and contraction were used. Both signal periods were manually and visually checked, and quasi-stationarity ensured regarding the amplitude within each respective period. Furthermore, artefact afflicted episodes were excluded from analysis. The raw data were filtered (Butterworth, 30th order, cut-off at 2 and 250 Hz), rectified and smoothened before separation into two analysis windows for (a) muscle resting state and (b) isometric contraction state. The analysis epochs were used for further analysis. The rectified and filtered sEMG signals also showed that the recorded amplitudes are in the order of hundreds of μV to very few mV.

One of the most common approaches used to calculate the signal characteristics in frequency domain is the power spectral density (PSD). Calculation of the mean PSD allows to estimate the power of the signal at different frequencies, being one of the most used approaches to investigate sEMG data. So, PSD is a useful tool to compare the signal quality of the different dry electrodes in the frequency domain, and also to understand how they behave when compared to conventional ones. Moreover, although the analysis of the PSD spectra is useful to evaluate the performance of the electrodes in the frequency domain, a well-known approach for a direct signal quality assessment is the calculation of the signal to noise ratio (SNR), e.g., using the sEMG amplitude during contraction versus the unwanted electrical signal noise during muscle relaxation. Accordingly, the higher the SNR, the better the signal acquired by the electrodes.

Related information about detailed signal processing can be found in the literature [33,34,35,36,37,38].

## 3. Results

### 3.1. Morphological, Structural and Electrical Characterization of the Thin Films

**Figure 3:**
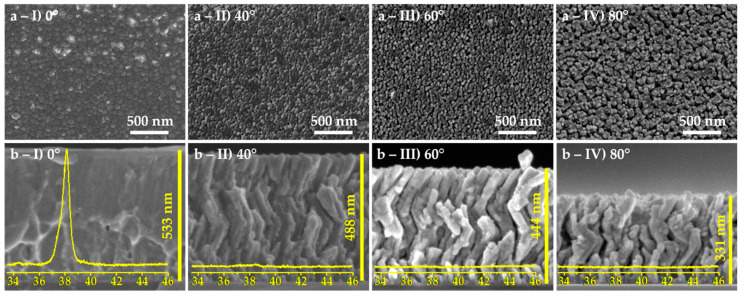
Scanning electron microscopy images of the thin films: (**a**) top-view and (**b**) cross-section micrographs, and the corresponding X-ray diffractograms for the different incidence angles, (I) 0°, (II) 40°, (III) 60°, and (IV) 80°.

**Figure 4:**
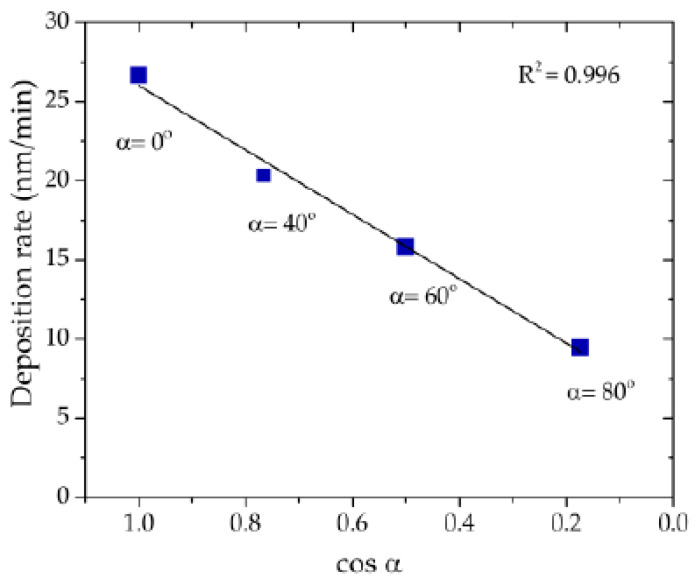
Deposition (growth) rate of the thin films as a function of the cos α (α: incidence angle), following the cosine law tendency.

The microstructure of the thin films can be observed in Figure 3, for the different α angles. Top-view images, Figure 3a, were used to evaluate the influence of the incidence angle α on the surface porosity of the thin films. The cross-section images, Figure 3b, show (I) the thin film deposited in conventional angle, and (II to IV) the zigzag architectures obtained for incidence angles α of 40°, 60°, and 80°. The thicknesses of the thin films are also labelled in Figure 3. These values were used to determine the corresponding deposition rates, which are plotted in Figure 4, following the typical cosine law tendency [39]. The thin film deposited with incidence angle α = 0°, presents an almost featureless growth microstructure. Moreover, the XRD pattern (also included in Figure 3b-I) shows a diffraction peak corresponding to the (002) orientation of the hcp structure of Ti (ICSD collection code # 44872). When the α angle increases, there are morphological changes that stand out. The zigzag microstructure is already observed at α = 40° and the film seems to become more porous for higher α angles, an evolution that agrees with the top-view micrographs. Moreover, the structural analysis made by XRD shows a loss of crystallinity for all zigzag films.

**Figure 5:**
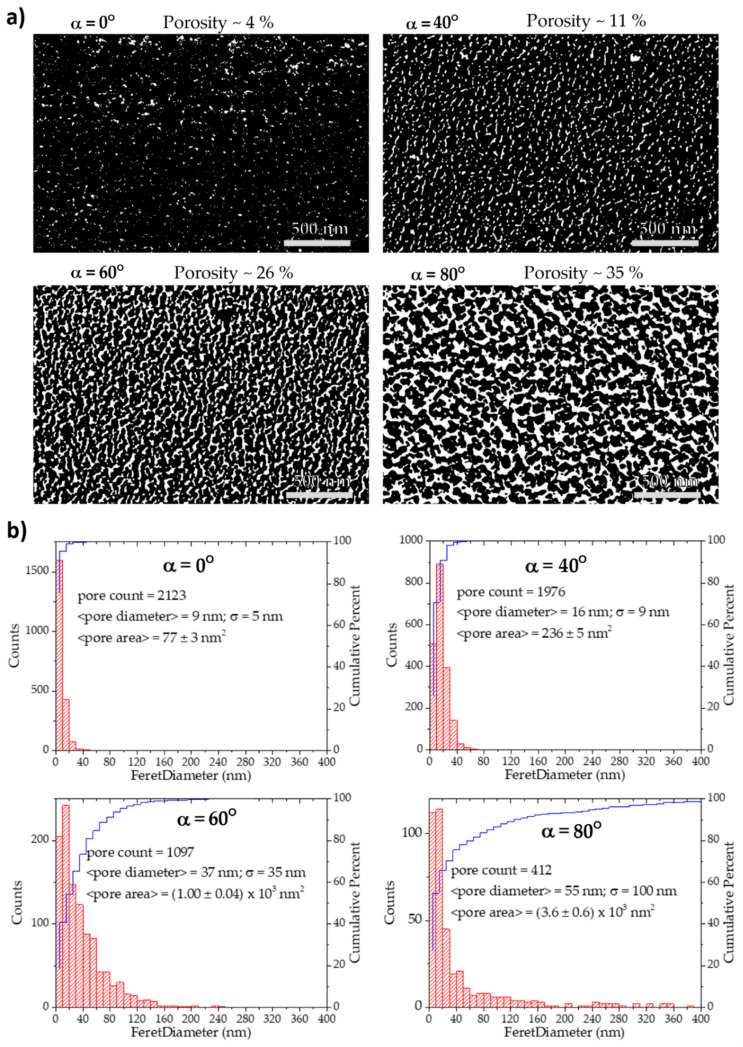
(**a**) Top-view SEM micrographs processed in MATLAB for surface porosity calculation and (**b**) corresponding histograms.

The surface porosity of each Ti microstructure was analyzed using an algorithm implemented in MATLAB [27], by producing binary images using a thresholding approach. The results are presented in Figure 5, showing a considerable increase in the surface porosity (white areas) with the rise of the incidence, α, angle (Figure 5a), from 4% (at α = 0°) up to 35% (at α = 80°). Besides the porosity calculation, the pores were analyzed and their Feret diameter distribution is presented in Figure 5b, as well as the pore count and their average area.

It is obvious from the analysis of the histograms (Figure 5b) that the number of pores is initially low, but growing, and their size distribution is becoming wider as the incidence angle increases. The average pore Feret diameter progressively increases from 9 nm (standard deviation of the distribution, σ = 5 nm) at α = 0°, to 16 nm (σ = 9 nm) at α = 40°, then to 37 nm (σ = 35 nm) at α = 60°, ending up in 55 nm (σ = 100 nm) when the films are deposited with the highest angle, α = 80°. The average individual pore area, increases from an incidence angle of 0° (77 nm^2^) by 3 times at α = 40° (236 nm^2^), 13 times at α = 60° (1.00 × 10^3^ nm^2^), and almost 50 times at α = 80° (3.6 × 10^3^ nm^2^). Therefore, beyond an evident evolution of the surface porosity, it is also noteworthy that the type of porosity is evolving. At α = 0°, the thin film can be classified as microporous since the pores are of a few nm (using IUPAC’s recommendations [40]). For an incidence angle of α = 40°, the number of micropores is reduced, giving rise to mesoporosity, as most parts of the pores have sizes between 2 nm and 50 nm. At α = 60°, the thin film is essentially mesoporous, yet the number of macropores (width larger than 50 nm) is significant. Finally, when the thin film is deposited with α = 80°, wider pores appear and the film can be globally classified as macroporous, although mesopores are still present.

**Figure 6:**
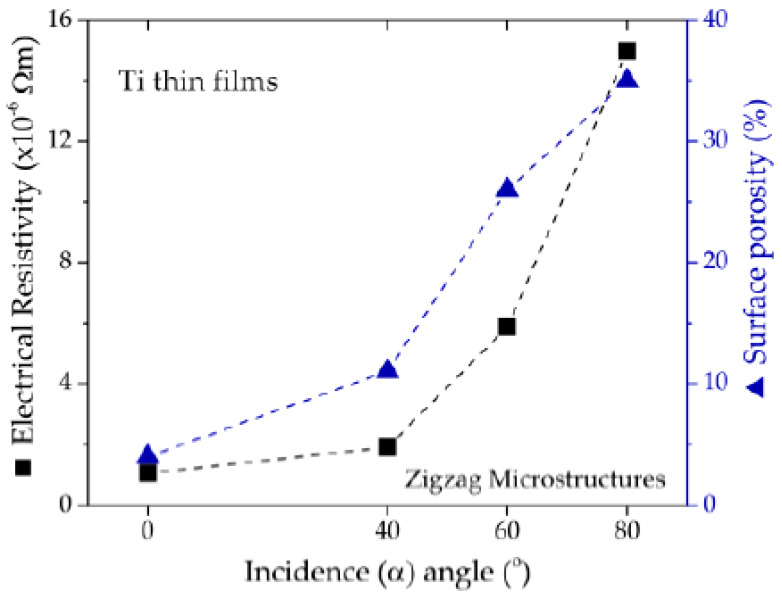
Electrical resistivity and surface porosity of the thin films as a function of the incidence α angle.

The values of the electrical resistivity of the thin film shown in Figure 6 follow a nearly exponential behavior with increasing α angle. The electrical resistivity of the zigzag thin film deposited with an incidence angle of 80° is remarkably higher (about 15 times) than the resistivity of the thin film deposited with an incidence angle 0°. The surface porosity follows a similar trend as also depicted in Figure 6. As expected, the increase of the incidence sputtering angle produces grain boundaries, which are barriers for electrons’ conduction, and progressively enlarges those discontinuities in the film, inducing the enhancement of the electrical resistivity [41].

### 3.2. Electrodes’ Characterization

#### 3.2.1. Electrode–Skin Impedance

Considering the microstructural and electrical properties of the prepared thin films, the ones prepared at incidence angles α = 0° and α = 60°, and deposited onto SS and TPU substrates were selected for further biosignal acquisition tests in volunteers. This selection allowed the comparison of a “conventional” thin film microstructure (α = 0°), with reduced porosity, with a nearly mesoporous thin film (α = 60°), i.e., with a higher electroactive surface area, without compromising a good electrical conductivity (see Figure 6). Table 1 summarizes the main characteristics of the samples used in the in-vivo tests.

The electrode–skin contact plays an important role in the quality of a biosignal acquisition. To obtain good SNR values in sEMG recordings it is fundamental to ensure relatively low and stable impedance values at the electrode–skin interface. The electrode–skin impedance was measured at different frequencies (10 Hz to 1000 Hz), allowing the comparison between the conventional gel-based Ag/AgCl electrodes and the Ti-based dry-contact electrodes. Although the energetic distribution of an EMG signal is basically within the frequency interval of 10–500 Hz, with the dominant components in the 50–150 Hz range, wider ranges of test signal frequencies are important to understand the electrode’s behavior at lower and higher frequencies.

**Figure 7:**
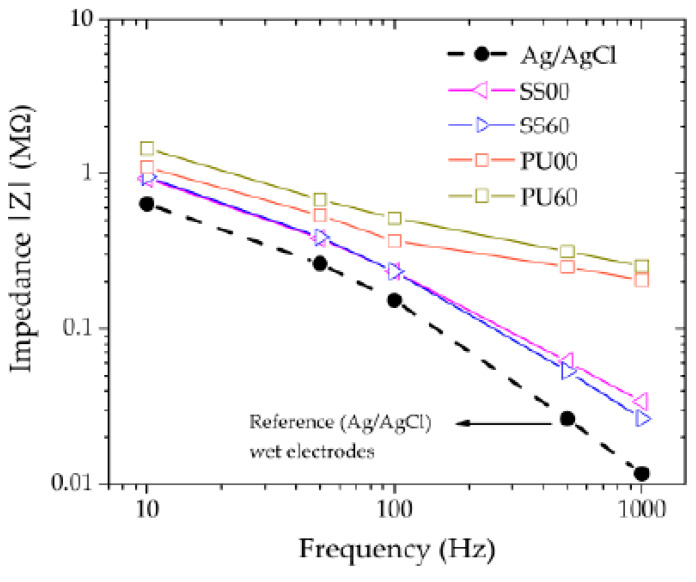
Grand average impedance values of the five measurement frequencies and the different electrodes calculated over all volunteers.

Figure 7 shows the grand average absolute impedance values (calculated from all volunteers) of the tested electrodes, measured for the discrete frequencies. The Ag/AgCl electrodes have the lowest impedance values over all frequencies, tending to a value of about 10 kΩ, as expected. The impedance values of the SS electrodes are of the same order of magnitude as the Ag/AgCl reference electrodes, and there are no significant differences between both microstructures (α = 0° and 60°) in the frequency band between 10 Hz and 1000 Hz. Moreover, SS electrodes follow nearly the same trend as the reference electrodes of Ag/AgCl with increasing frequency. The electrode–skin impedance of both TPU electrodes was slightly higher than for SS electrodes, for frequencies up to 100 Hz. For higher frequencies, the difference is larger, and TPU electrodes’ impedance tend to values around 200–250 kΩ, which are one order of magnitude higher than SS electrodes (and reference electrodes).

Traditionally, clinical and neuropsychological standards pose a rigid requirement on electrode-skin impedances in the order of 10 kΩ in order to minimize the related impedance effects [42]. However, modern signal amplifiers provide considerably improved dynamic input impedances up to the GΩ range [43]. This considerably reduces the influence of electrode–skin impedance level and homogeneity on the signal quality and noise, thus enabling the use of dry electrodes exhibiting electrode–skin impedances up to the range of a few MΩ. The impedance values registered for the dry-contact TPU electrodes are still well below the limits reported in the literature.

Both TPU and SS electrodes, functionalized with Ti thin films, were used for sEMG recordings and the results compared with the reference electrodes of Ag/AgCl.

#### 3.2.2. In-Vivo sEMG Acquisition

**Figure 8:**
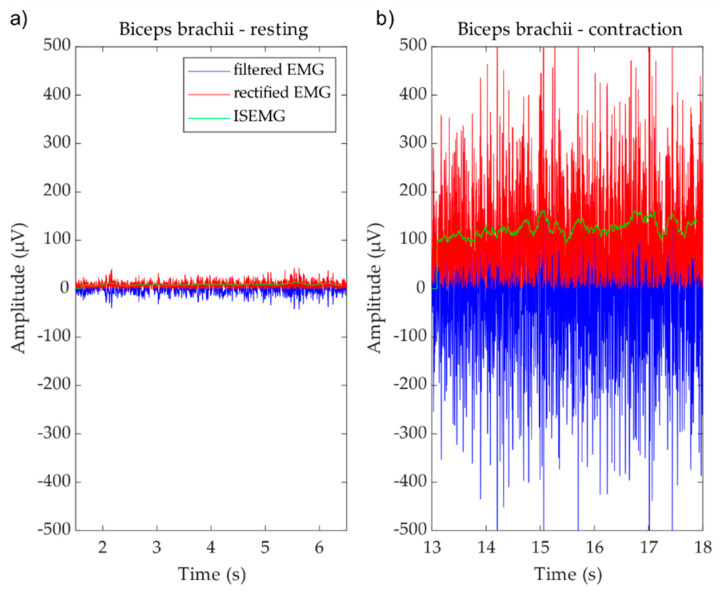
Example of one sEMG recording on the Biceps brachii muscle. Example of filtered (blue), rectified (red), and average smoothed sEMG (green) recording for (**a**) resting and (**b**) contraction states.

After the measurements of impedance values, sEMG biosignals of the same group of volunteers were acquired. As aforementioned, raw data were filtered, rectified, and smoothened before separation into two analysis windows for (a) muscle resting state and (b) isometric contraction state, as is visible in Figure 8. The recorded amplitudes are in the order of hundreds of μV to very few mV, which are typical amplitudes for EMG signals [43,44].

**Figure 9:**
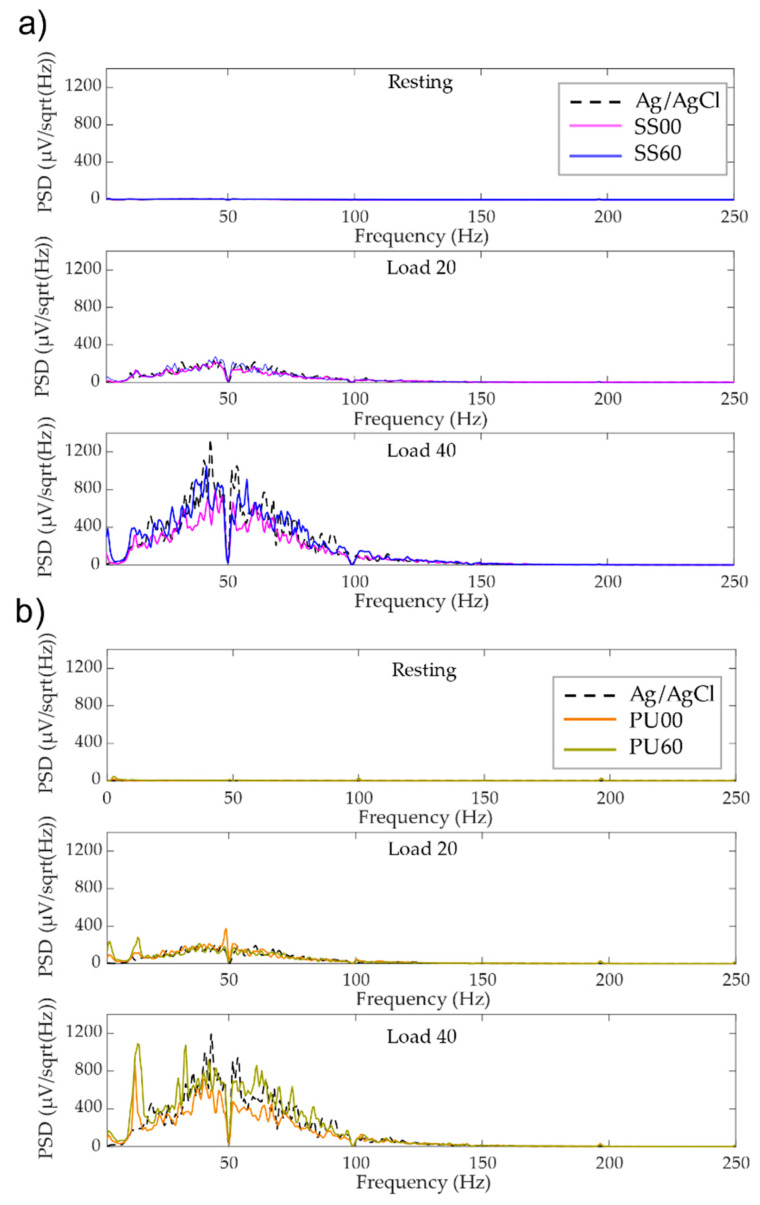
Power spectral density (PSD) grand average of the tested (**a**) stainless-steel (SS) and (**b**) thermoplastic polyurethane (TPU) electrodes and reference electrodes of the processed signal segments.

Figure 9 shows the PSD profiles of the processed signal segments calculated using the Welch estimation method [45] applied to the aforementioned analysis episodes. The PSDs of all electrodes show the expected characteristics and the frequency band of the sEMG ranges from 10 Hz up to 250 Hz.

The PSD considerably increases within the investigated frequency range for both tested electrode types (SS and TPU), following the Ag/AgCl reference electrodes response, when the contraction load rises from 20% (load20) to 40% (load40). The drop of power around 50 Hz, observed in all the cases, is caused by the applied powerline filter.

Analyzing the PSD profile of the SS dry electrodes one can report that the Ti thin film zigzag microstructure (α = 60°) behaves better for load40 as it originates power signals closer to the conventional electrodes (Figure 9a). For the lowest load (load20), PSD profiles are rather similar. Regarding TPU electrodes’ performance, the lowest load (load20) maintains a regular behavior, almost matching conventional electrodes. Nevertheless, for 40% load the PSD deviates from the Ag/AgCl electrodes. Noteworthy are the additional power peaks below 20 Hz observed for these electrodes, which might be associated to other sources of noise [15]. These artefacts could not be extracted from the signal, but fortunately, are out of the typical range of interest for EMG signals.

**Figure 10:**
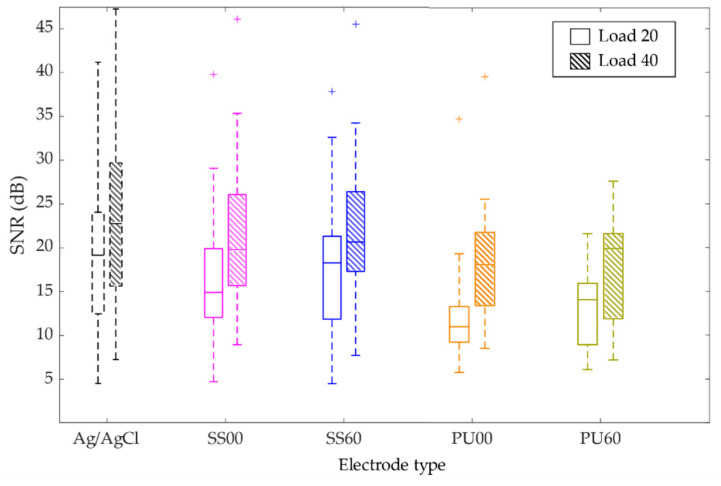
Signal-to-noise ratio (SNR) of the tested SS and TPU electrodes for the load of 20% and 40% of the volunteer’s maximum load.

Figure 10 show boxplots where each distribution includes data from one set of dry electrodes re-used in all volunteers. In the case of the reference electrodes of Ag/AgCl one set per volunteer was used since these are disposable.

From Figure 10 it is obvious that for all electrode types the SNR significantly increases from load20 to load40. For load20, Ag/AgCl shows the highest values (median = 19.2 dB). Compared to this reference value, SS60 is the dry electrode with the closest value (median = 18.3 dB), while PU00 electrode shows the lowest value (median = 11.0 dB). Noteworthy is the clear improvement of SNR values when the thin films’ microstructure changes from normal incidence (α = 0°) to zigzag microstructures (α = 60°).

For load40 the SNR values of Ag/AgCl and dry electrodes are tendentiously higher. The reference here is a median of 22.8 dB, for Ag/AgCl electrodes. The medians of the remaining electrodes range between 20.7 dB for SS60 (the higher among dry electrodes, for load40) and 18.0 dB PU00 (the lowest, for load40), thus confirming that the electrodes prepared with films deposited at an oblique angle (α = 60°) show better performance than the films deposited at normal incidence (α = 0°). Another important feature is that although TPU electrodes have lower SNR values than SS electrodes, they show lower variability, even when compared to the reference electrodes.

## 4. Discussion

In this work, novel types of dry electrodes for sEMG were developed. They are based on Ti thin films deposited on SS and TPU substrates using oblique angle deposition to produce zigzag-like microstructures. The architectured thin films have been assessed in terms of morphological, structural, and electrical characteristics, followed by a study about their performance as sEMG electrodes in an in-vivo study in twenty volunteers. The prepared dry electrodes were always compared to conventional wet Ag/AgCl electrodes in terms of impedance values and sEMG performance.

The investigation of the thin films’ morphology showed that the zigzag microstructures were achieved for incidence angles between α = 40° and α = 80°. The obtained microstructures induced porosity, hence increasing the electrical resistivity. Among the prepared zigzag microstructures, the thin film deposited at α = 60° was found to hold a good compromise between the microstructural characteristics and the electrical behavior, and it was selected for further studies related to sEMG recordings. These zigzag architectures, nearly mesoporous, and also the type of substrate (SS and TPU), affected the electrode–skin impedance and therefore the signal quality of sEMG recordings.

During the initial application and subsequent recordings, the handling of the electrodes with the deposited thin films (in SS and TPU) was perceived simpler by all operators and volunteers, than the handling of wet Ag/AgCl electrodes. The use of Ag/AgCl electrodes caused pain during the removal and left marks on the patients’ skin, as being described in the literature [5,9]. These marks, which in some cases last for several days, might be a sensitive or even allergic reaction to the electrolyte gel combined with the adhesive, since they appeared in the interface between both. Several days after the acquisitions, volunteers reported to still have smooth skin irritations resulting from the wet Ag/AgCl electrodes, whereas the dry electrodes did not cause any long-lasting mark. It is worth to mention that the reference Ag/AgCl electrodes were changed for each volunteer, while the dry electrodes were reusable, and only two pairs of each were necessary.

The dry electrodes were cleaned with ethyl alcohol (70% v/v), after each utilization for sEMG acquisitions. In particular, the surface of the electrodes deposited with an incidence angle of α = 60° needed to be carefully dried with a dry cloth, otherwise the alcohol caused stripes on the thin film. This effect is likely to be related to the effect of the microstructure and porosity of the thin film.

One of the reasons for the differences observed in the performance of TPU and SS electrodes is related to the less stable electrode-wire connection in TPU electrodes due to the electrical insulating nature of the polymer. Moreover, it also caused increased sensor impedance and is likely the reason for higher interference levels, especially for load40, which could not be filtered. This means that these electrodes may be more susceptible to noise and motion artefacts. Thus, allied to a further improvement of the physical properties of the thin film electrodes, it is of importance to improve the mechanical and electrical connection between cabling and electrodes, increasing stability and reliability of the sEMG recordings.

## 5. Conclusions

A major conclusion that can be drawn from this work is that the signal processing was successful to compare the developed dry electrodes against the reference Ag/AgCl electrodes. Dry electrodes were prepared by sputtering deposition of architectured Ti thin film onto thermoplastic polyurethane (TPU) and stainless-steel (SS) substrates. These dry electrodes have shown better usability and improved biocompatibility properties during in-vivo tests. It was demonstrated that the signal quality was improved when zigzag thin films (with nearly mesoporous porosity) were used for the sEMG recordings. Further optimization of deposition parameters and processes could probably improve the electrical performance of the thin film architectures on polymeric substrates. The results emphasize the utility and advantages of dry electrodes for sEMG recordings and can support increased use of sEMG in clinical and research applications.

## Figures and Tables

**Table 1:** Reference of the selected electrodes tested on each volunteer.

Reference	Electrode Base	Ti Thin Film
Ag/AgCl	Conventional Ag/AgCl	---
SS00	Stainless Steel	0°
SS60	Stainless Steel	60° zigzag
PU00	Thermoplastic Polyurethane	0°
PU60	Thermoplastic Polyurethane	60° zigzag
